# A devastating blow: personal reflections on Argentina’s scientific decline

**DOI:** 10.1128/jvi.00549-24

**Published:** 2024-04-16

**Authors:** Daniel R. Perez

**Affiliations:** 1 Department of Population Health, College of Veterinary Medicine, University of Georgia, Athens, Georgia, USA; The University of Arizona, Tucson, Arizona, USA

**Keywords:** funding, Argentina, science, policy

## Abstract

As an Argentine scientist, the defunding of CONICET and INTA feels like a blow to progress and our future. Despite free education, these cuts force talented researchers to seek opportunities abroad. Argentina’s history of scientific achievement, from Nobel Prizes to COVID-19 vaccines, is at risk. Defunding science weakens our ability to solve problems and compete globally.

## COMMENTARY

As a scientist who honed my skills in Argentina, the recent government decision to defund the National Scientific and Technical Research Council (CONICET) and the National Institute of Agricultural Technology (INTA) feels like a personal gut punch. It is a devastating blow not just to scientific progress, but to Argentina’s future.

A quick search on PUBMED with “virus OR parasite OR bacteria” alongside “CONICET” or “INTA” reveals over 12,000 peer-reviewed publications in prestigious journals. That is a testament to the relentless pursuit of knowledge by these institutions. But the impact goes beyond numbers—it is the talent we lose.

Argentina boasts free college education, a privilege many countries lack. Yet, by cutting science funding, we are essentially throwing that advantage away. Why invest in educating bright minds if we cannot offer them the resources to thrive in scientific fields? This short-sightedness only fuels the “brain drain.” Young, talented researchers, myself included in 1990, are forced to seek opportunities abroad, often with little incentive to return.

CONICET, with its apolitical research centers, has propelled Argentina to the forefront of scientific discovery. Economic challenges are undeniable, but slashing science funding is akin to throwing away the key to future progress.

Think about it—Argentina boasts three Nobel laureates in science: Bernardo Houssay, Luis Leloir, and César Milstein. These are just a few of the countless minds that have positioned Argentina as a scientific powerhouse.

Stifling this vibrant research ecosystem hurts everyone. From groundbreaking cancer research to advancements in agriculture, Argentine science has demonstrably benefited the world. Defunding it weakens Argentina’s ability to tackle its problems, from poverty reduction to technological advancement.

The recent cuts to fellowships, promotions, and even administrative staff at CONICET are gutting the very foundation of a thriving scientific community. This is a community that has developed its own COVID-19 vaccine, launched groundbreaking communication satellites, and even built next-generation nuclear reactors. These achievements would not have been possible without sustained government support.

Let us not forget another crucial aspect: Argentina struggles with poverty, with estimates suggesting over 40% of the population living below the national poverty line (source World Bank). Meanwhile, high school students in Argentina scored less than the average in mathematics, reading, and science in standardized tests like PISA (Program for International Student Assessment). Investing in science is not a luxury; it is a pathway to solving these issues. Investing in science education is paramount to nurturing the next generation of researchers and innovators. Scientific advancements lead to innovations that create jobs, improve healthcare, and ultimately elevate living standards.

Destroying a system built over decades will inflict lasting damage. It will lead to an exodus of young minds, further deepening the social divide and hindering Argentina’s ability to compete on the global stage. Let us work together to ensure Argentina’s scientific torch continues to shine bright. The future depends on it.

